# BIOLOGICAL AGING PATHWAYS THAT UNDERLIE LIFE COURSE SOCIOECONOMIC DISPARITIES IN PHYSICAL FUNCTION AND LONGEVITY

**DOI:** 10.1093/geroni/igae098.2204

**Published:** 2024-12-31

**Authors:** Tara Gruenewald, Naomi Podber

**Affiliations:** Chapman University, Orange, California, United States; Chapman University, Orange, California, United States

## Abstract

The poorer functioning and greater morbidity and mortality risk in those with greater life course socioeconomic disadvantage has led to the search for the biological processes that underlie these associations, including accelerated cellular and system aging. Our study examines whether a DNA methylation profile of the pace of biological aging (DunedinPACE) and a multi-system index of physiological dysregulation (allostatic load (AL)) mediate associations between life course socioeconomic disadvantage (SESDIS) and both physical function (gait speed, chair stand time) and survival. Data are from the Midlife in the U.S. Study (N=1,295, age 26–86, 55.4% female, 69.0% white). Measures included a multi-indicator composite of childhood and adult socioeconomic indicators (SESDIS), DunedinPACE as described in Belsky et al. (2022), AL as described in Gruenewald et al. (2012), gait speed (time to complete a 50-foot walk), and chair stand time (5 consecutive rises from a seated position). Analytic models included z-scored predictors, age, race, and gender covariates and accounted for family clustering. Greater SESDIS was associated with increased mortality hazard (HR=1.42***), higher gait speed (b=0.70***) and chair stand time (b=0.85***), and higher DunedinPACE (b=0.27***) and AL (b=0.19***). Greater DunedinPACE and AL were associated with slower gait and chair stand time and lower survival. Multi-mediation analyses indicated significant bootstrapped (1,000 replications) indirect effects for DunedinPACE and AL as mediating pathways of the slower gait and chair stand time and lower survival in those with greater SESDIS. Findings highlight biological aging pathways that may contribute to life course socioeconomic disparities in physical function and longevity.

